# Distinct fungal communities associated with different organs of the mangrove *Sonneratia alba* in the Malay Peninsula

**DOI:** 10.1186/s43008-020-00042-y

**Published:** 2020-09-15

**Authors:** Nicole Li Ying Lee, Danwei Huang, Zheng Bin Randolph Quek, Jen Nie Lee, Benjamin J. Wainwright

**Affiliations:** 1 Department of Biological Sciences, National University of Singapore, 16 Science Drive 4, Singapore, 117558 Singapore; 2 Tropical Marine Science Institute, National University of Singapore, 18 Kent Ridge Road, Singapore, 119227 Singapore; 3 Faculty of Science and Marine Environment, Universiti Malaysia Terengganu, 21030 Kuala Nerus, Malaysia; 4 Yale-NUS College, National University of Singapore, 16 College Avenue West, Singapore, 138527 Singapore

**Keywords:** Fungal diversity, Internal transcribed spacer, Mangrove microbiome, Marine fungi, Microbial ecology, Southeast Asia

## Abstract

Mangrove forests are key tropical marine ecosystems that are rich in fungi, but our understanding of fungal communities associated with mangrove trees and their various organs remains limited because much of the diversity lies within the microbiome. In this study, we investigated the fungal communities associated with the mangrove tree *Sonneratia alba* throughout Peninsular Malaysia and Singapore. At each sampling location, we collected leaves, fruits, pneumatophores and sediment samples and performed amplicon sequencing of the ribosomal internal transcribed spacer 1 to characterise the associated communities. Results show distinct fungal communities at each sampled location with further differentiation according to the plant part. We find a significant distance decay of similarity, particularly for sediment samples due to the greater variability of sediment environments relative to the more stable fungal habitats provided by living plant organs. We are able to assign taxonomy to the majority of sequences from leaves and fruits, but a much larger portion of the sequences recovered from pneumatophores and sediment samples could not be identified. This pattern underscores the limited mycological research performed in marine environments and demonstrates the need for a concerted research effort on multiple species to fully characterise the coastal microbiome and its role in the functioning of marine ecosystems.

## INTRODUCTION

Mangroves are a globally distributed group of salt tolerant trees and shrubs that are confined to brackish intertidal zones, estuaries, lagoons and backwaters throughout the tropics and subtropics (Thatoi et al. 2013). Straddling the interface between marine and terrestrial ecosystems, they provide important buffers that dissipate wave energy, stabilising coastlines and protecting against coastal erosion and natural hazards such as hurricanes and tsunamis (Williams 2005; Wee et al. 2019). Mangroves provide critical ecological habitats that host high levels of biodiversity and act as a nursery for many juvenile coral reef species (Abu El-Regal and Ibrahim 2014; Mehvar et al. 2018). Despite these benefits, mangrove clearance for aquaculture and urban development is rampant in many areas of the world. As much as 35% of global mangrove cover has been removed, with Asia having lost an estimated 33% of its total mangrove cover between 1980 and 1990 (Richards and Friess 2016; Sanderman et al. 2018).

Mangroves are acknowledged as biodiversity hotspots of marine fungi (Shearer et al. 2007). Pioneering mycological research documented fungi on mangrove roots (Cribb and Cribb 1955) and described mangrove-associated fungi throughout the world (Kohlmeyer 1969). New species continue to be described today, and examination of various plant parts and geographic localities are finding unexpected diversity and strong community structuring of fungi in mangroves (Kumar et al. 2019; Lee et al. 2019a). In particular, mangroves abut marine and terrestrial environments, offering a unique opportunity to study plants that potentially contain obligate marine fungi, terrestrial fungi and those that can survive, or at least tolerate both environments to some degree. Mangrove-associated fungi have been divided into two groups: those that are submerged at high tide and those that are not. Marine fungi are expected to be found in parts that can be submerged, while terrestrial fungi are likely to dominate parts that are not, or are unlikely to be submerged (i.e. leaves and fruits) (Kohlmeyer 1969; Lee et al. 2019b).

Studies on marine fungi remain sparse despite the recent interest, particularly those examining biogeographic patterns (Amend et al. 2012, 2019; Wainwright et al. 2017, 2018; Ettinger and Eisen 2019), which is unfortunate as marine systems offer a potential treasure trove of undescribed marine fungal biodiversity. In 2011, only 537 obligate marine fungi had been identified (Jones 2011). Today, estimates predict in excess of 10,000 marine species, and nearly all remain undescribed (Jones 2011; Comeau et al. 2016; Picard 2017; Amend et al. 2019; Jones et al. 2019). Consequently, our understanding of fungal biogeography in marine environments remains rudimentary. However, the research that has been performed suggests that the environment strongly influences spatial patterns of marine fungal communities (Tisthammer et al. 2016; Lee et al. 2019a). Environmental and habitat differences also explain differences in seagrass and macroalgal associated fungal communities in Southeast Asia (Wainwright et al. 2019b, c).

These discoveries are occurring during a time of growing interest and appreciation of the marine microbiome and how this is influenced by, or influences the microbiome of species found in coastal areas (Glasl et al. 2019a, b; Trevathan-Tackett et al. 2019). Yet, there remains a lack of research into the coastal microbiome and this is especially apparent in comparison to most other microbiome types (e.g., the human microbiome) (Trevathan-Tackett et al. 2019; Wilkins et al. 2019). Specifically, despite the recognised necessity of mangroves and the critical ecosystem services they provide, work on the mangrove microbiome is embryonic at best, especially in contrast to the more charismatic coral reefs (Buddemeier and Smith 1999) where studies are relatively numerous and advanced in comparison (Ainsworth and Gates 2016; Hernandez-Agreda et al. 2017; Gardner et al. 2019; Wainwright et al. 2019a). For example, there are efforts to engineer, manipulate and seed the coral microbiome with beneficial microorganisms that could promote recovery from disturbance (Peixoto et al. 2017, 2019; Rosado et al. 2019).

Here, using high-throughput sequencing, we examine fungi associated with the mangrove tree *Sonneratia alba* throughout the Malay Peninsula to test whether fungal communities differ by geographic location and plant part sampled. Together with samples of the adjacent sediment, we provide valuable information on the fungal composition of the *S. alba* microbiome and its associated environment.

## MATERIALS AND METHODS

**Fig. 1:**
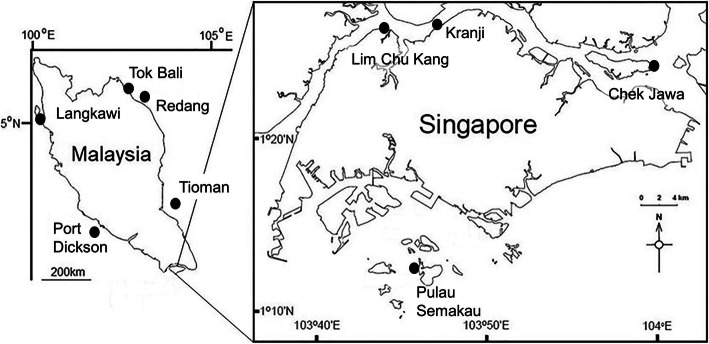
Map of sampling locations throughout Singapore and Peninsular Malaysia

We targeted 10 visibly healthy whole leaves, fruiting bodies (mangrove fruit) and entire pneumatophores from *Sonneratia alba* trees during low tide from each of nine locations in three regions (Singapore, western and eastern Peninsular Malaysia), though fewer samples were collected in some cases due to logistical or safety reasons (i.e., collecting fruits and leaves at height) (Fig. 1 and Supplementary Table 1). Additionally, one sediment sample in close proximity (< 1 m) to each tree was taken using a syringe placed approximately 4 cm below the surface. Prior to DNA extraction, 0.5-cm diameter leaf-disks were taken throughout the surface of the leaf with a sterile hole punch. Pneumatophores and *S. alba* fruiting bodies were cut into ~ 0.25 cm cubes using a new sterile razor blade for each sample. All collected mangrove tissues (leaves, fruits and pneumatophores) were surface sterilized by immersion in 1% NaClO for 2 min, 70% EtOH for 2 min and rinsed twice in sterile, autoclaved water for 5 min. Sediment samples were not surface sterilized. Tissue and sediment samples were disrupted in an Omni Bead Ruptor 24 (Omni International, Kennesaw, GA, United States) at 8 ms^− 1^ for 2 min.

As per Cobian et al. (2019), haphazardly chosen surface sterilized tissues were used in DNA extractions, and all extractions were performed using the Qiagen DNeasy PowerSoil Kit following the manufacturer’s protocol. The internal transcribed spacer 1 (ITS1) region of fungal DNA was amplified via polymerase chain reaction (PCR) using the ITS1F primer (5′-CTT GGT CAT TTA GAG GAA GTA A-3′ (Gardes and Bruns 1993) and the ITS2 primer (5′-GCT GCG TTC TTC ATC GAT GC-3′ (White et al. 1990). Primers were modified to include Illumina adapters, a linker and a unique barcode (see Smith and Peay 2014 for details of custom sequencing primers). Each reaction was performed in a total volume of 25 μl, containing 12.5 μl KAPA Plant PCR buffer, 1.5 μl BSA, 0.5 μl MgCl2, 0.1 μl KAPA 3G Plant DNA polymerase (Kapa Biosystems, Inc., Wilmington, MA, United States), 0.75 μl of each primer at 10 mM, and 9 μl DNA template. PCR cycling parameters were: 3 min at 95 °C, followed by 35 cycles of 20 s at 95 °C, 15 s at 53 °C, and 20 s at 72 °C, with a final elongation at 72 °C for 1 min. Negative PCR and extraction blanks were included and sequenced to identify contamination issues. PCR products were visualized on a 1% TBE buffer agarose gel, then normalized and cleaned using SequalPrep™ normalization plates (Invitrogen, Frederick, MD, United States). Purified PCR products were sequenced on the Illumina MiSeq platform (600 cycles, V3 chemistry, 300-bp paired-end reads) with a 15% PhiX spike at the Genome Institute of Singapore.

Our bioinformatics pipeline, comprising quality filtering and taxonomic assignment, followed that described in the DADA2 ITS Pipeline Workflow V1.81 (https://benjjneb.github.io/dada2/ITS_workflow.html), with the following minor modifications: (1) due to lower quality, reverse reads were not used – discarding low quality reverse reads is a common strategy that frequently gives better results than assembled reads (Pauvert et al. 2019); and (2) the R package *decontam* was used to identify and remove any contaminants identified in sequenced negative controls via the prevalence method (Davis et al. 2018).

All amplicon sequence variants (ASVs) not assigned to fungi were removed, while those remaining were used in all downstream analyses. Non-metric multidimensional scaling (NMDS) plots were created using a Bray–Curtis dissimilarity matrix of samples in the R package phyloseq version 1.25.2 (McMurdie and Holmes 2013). A NMDS plot was generated for all sampled compartments combined, and separate plots were implemented for each sampled plant organ (leaf, fruit, pneumatophore and sediment). Permutational multivariate analysis of variance (PERMANOVA) with 999 permutations performed via the *adonis* function in the R package vegan version 2.5–2 (Oksanen et al. 2019) was used to test the effects of region, location and plant part on the fungal communities. Venn diagrams were generated using the VennDiagram R package (Chen and Boutros 2011).

To test for distance decay of similarity, Mantel test was performed between geographic distance and community matrices using the *mantel.rtest* function in the ade4 package (Bougeard and Dray 2018) with 999 permutations. We also carried out multiple regression on distance matrices with 9999 permutations in the *ecodist* package.

All raw sequences associated with this work have been deposited at the National Center for Biotechnology Information under the BioProject ID PRJNA592423.

## RESULTS

**Fig. 2:**
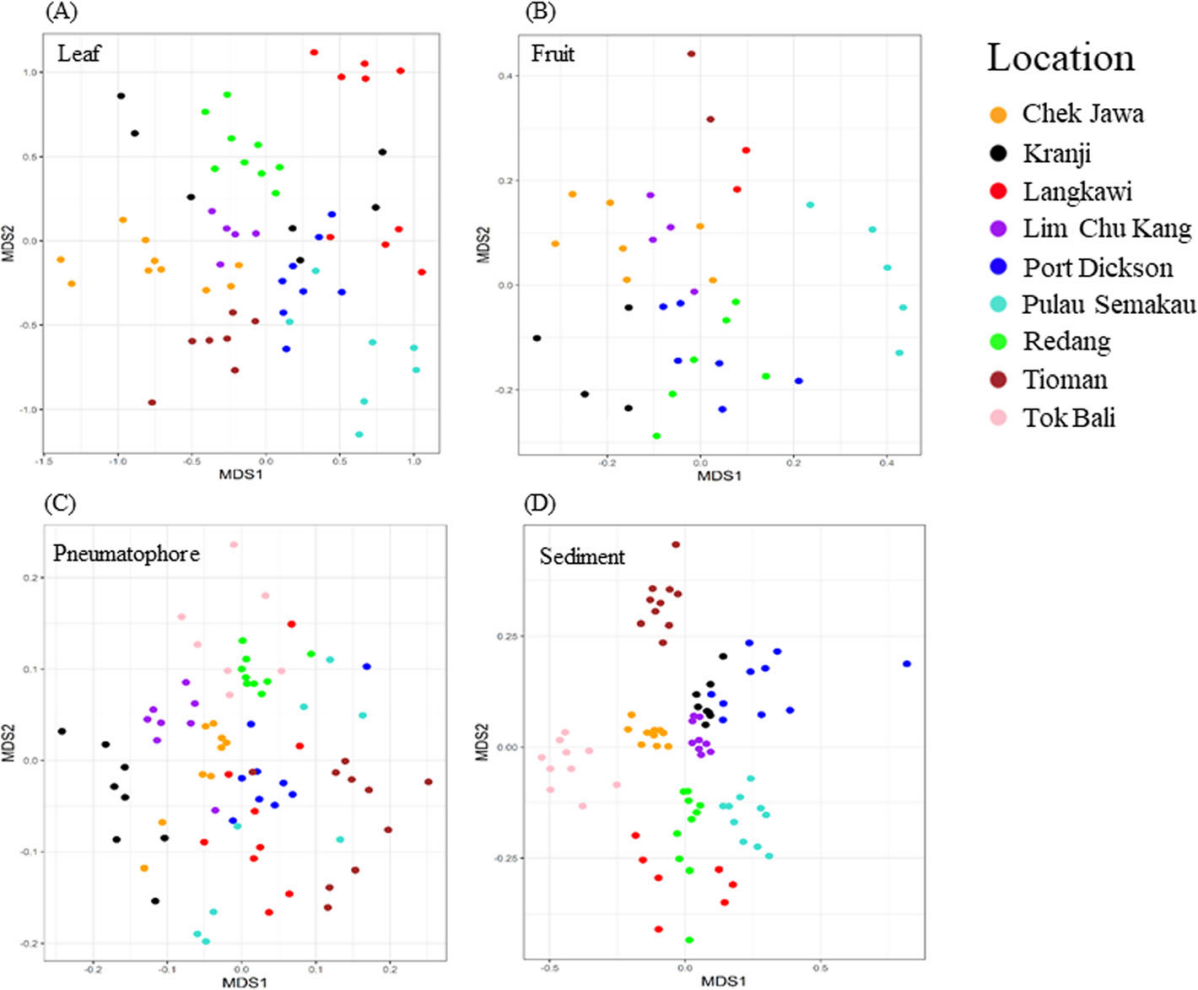
NMDS plots based on Bray–Curtis dissimilarity for each mangrove part and associated sediment sample, all show clear separation by location. **a** Leaf, Non-metric fit *R*^2^ = 0.948, linear fit *R*^2^ = 0.743, stress = 0.22 **b** Fruit, Non-metric fit *R*^2^ = 0.955, linear fit *R*^2^ = 0.775, stress = 0.21 **c** Pneumatophore, Non-metric fit *R*^2^ = 0.925, linear fit *R*^2^ = 0.618, stress = 0.23, **d** Sediment, Non-metric fit *R*^2^ = 0.946, linear fit *R*^2^ = 0.765, stress = 0.23

In total, 10,076,402 reads were generated, and after quality filtering, 5,417,789 were retained for downstream analyses. Rarefaction curves indicate sufficient sequencing depth was achieved (Supplementary Figure 1). NMDS plots show that fungal communities associated with each sampled compartment (leaf, fruit, pneumatophore and sediment) are clustered by location (Fig. 2). Fungal communities in sediment samples appear more similar to one another compared to the plant organs, and sediment samples are further clustered by region (Supplementary Figure 2). Overall, PERMANOVA indicates significant differences in fungal community among locations and sampled compartments (*R*^*2*^ = 0.116; *P* = 0.001 and *R*^*2*^ *=* 0.05; *P* = 0.001 respectively; Supplementary Table 2).

Structures that are not submerged at high tide (i.e., leaves and fruits) share more similar fungal communities than those that are periodically submerged by tides (Supplementary Figure 2). Supporting the idea that fungal communities from nearby sample locations are more similar to one another than distant ones, we see a significant positive relationship between community structure and geographic distance (Mantel test: *r* = 0.28, *p* = 0.001). This relationship is further supported by multiple regression on distance matrices for all sampled compartments combined (*r* = 0.07, *p* = 0.001) as well as for each compartment (Table 1).

**Table 1:** Mantel test and multiple regression on distance matrices (MRM) results for all compartments combined, and each individual compartment. All show a significant pattern of distance decay

	Mantel R statistic	Mantel Significance	MRM R^2^	MRM Significance
All	0.28	0.001	0.07	< 0.001
Fruit	0.23	0.001	0.07	< 0.001
Leaf	0.35	0.001	0.12	< 0.001
Pneumatophore	0.29	0.001	0.09	< 0.001
Sediment	0.50	0.001	0.21	< 0.001

**Fig. 3:**
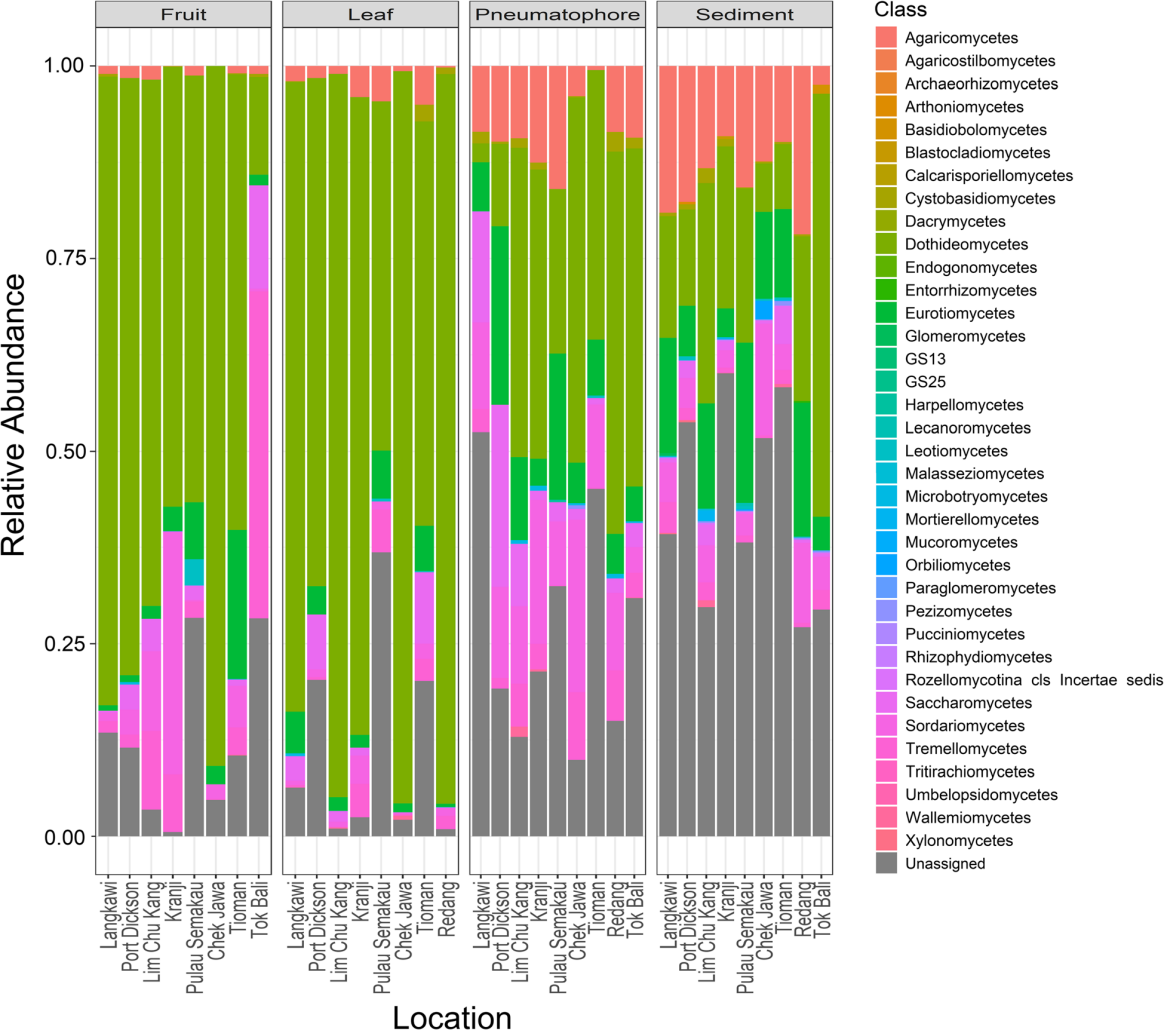
Stacked bar plots of relative class abundances in each part from each sample location

The most diverse fungal communities are associated with sediment samples which are approximately twice as diverse as all other sampled compartments, with median Shannon diversity values between 1.9 and 4.1 (Supplementary Figure 3). All samples, irrespective of type, are dominated by the phyla Ascomycota and Basidiomycota (Supplementary Figure 4) as well as the classes Dothideomycetes, Sordariomycetes and to a lesser extent the Eurotiomycetes, but the exact composition varies among compartments and locations (Fig. 3). When all organs and sediment samples are combined at each location, fungal diversity is relatively constant and composition at the class level is generally comparable throughout all locations (Supplementary Figures 5, 6 and 7).

**Fig. 4:**
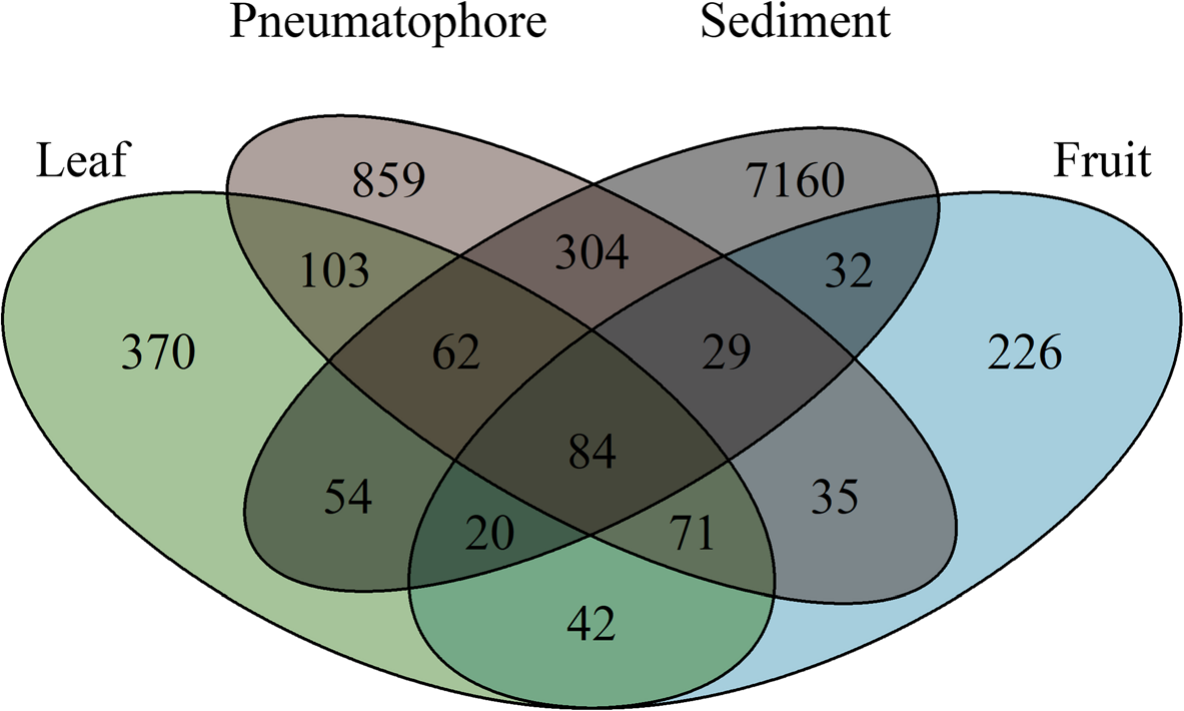
Venn diagram illustrating the number of amplicon sequence variants (ASVs) unique to each sampled part, and those shared between parts, showing that sediment has the highest fungal richness and 84 ASVs are shared between all sampled parts

Unique ASV richness is highest in the sediment, followed by pneumatophores (7160 and 859 respectively), while leaves and fruits—structures that are above the high water line—have the lowest unshared ASV richness with 370 and 226 respectively. A total of 84 ASVs are shared between all compartments including sediment, and the highest number of shared ASVs is found between pneumatophores and sediment samples (Fig. 4).

## DISCUSSION

In this study, we show that the fungal communities associated with the mangrove plant *Sonneratia alba* differ significantly between sampled locations throughout Singapore and Peninsular Malaysia. Fungal communities can be further differentiated by sampled compartment and plant organ (i.e., leaves vs. sediment, etc), with each compartment hosting a distinct fungal community. We also show a significant pattern of positive distance decay, meaning that fungal communities from geographically closer sites are more similar to one another than those that are more distant. This is consistent with work on a variety of other assumed highly dispersive taxa, from mammals to microorganisms, showing community or genetic structuring in the marine environment which has no obvious barriers to dispersal (Hoffman et al. 2012; Xu et al. 2017; Wainwright et al. 2018, 2019a, 2020). This contrasts with the traditionally held view that marine environments are open with few, if any limitations imposed on dispersal. Under this paradigm, highly dispersive taxa show limited structure of any nature (Rocha et al. 2007; Sá-Pinto et al. 2012), and from a microbial perspective as Baas Becking quipped “everything is everywhere” (Wit and Bouvier 2006).

As in Tisthammer et al. (2016), and also shown for another mangrove *Avicenna alba* recently (Lee et al. 2019a), we suggest that the fungal community distribution is strongly shaped by the environment, a consequence of differences in habitat type throughout Peninsular Malaysia and Singapore. Further supporting this idea, the coasts of Malay Peninsula are split in two by the Titiwangsa Mountain range, which forms the backbone of the peninsula with its > 2000-m maximum elevation. The east and west coasts have different tectonic origins and chemical compositions. The east coast is predominantly Carboniferous, while the west coast is dominated by Permian strata (Hutchison 2014). The compositional differences in strata create differences in substrate chemistry (e.g., organic, inorganic carbon content, carbon/nitrogen ratio and pH), and these differences can bring about environmental filtering that acts to remove members of the fungal community least suited to the environment (Cline and Zak 2014; Goldmann et al. 2016). Working in tandem with this is an environmental cline of increasing marine water mass salinity and dissolved oxygen with increasing latitude (Muhaimin et al. 2011). Consequently, locations close to one another are more similar in terms of fungal community composition than those more spatially separated, a result supported by Mantel test and multiple regression on distance matrices. All plant organs and sediment samples show significant patterns of distance decay, however, fungal communities associated with sediment display the strongest pattern of distance decay. This is consistent with previously proposed hypotheses suggesting that habitats offered by living plant organs (i.e., leaf, fruit, etc), while different between sampling regions, are more similar to one another and constant throughout a species range than would be suggested by environmental differences (Goldmann et al. 2016).

Shannon diversity values are comparable among all locations when all organs and sediment are combined, and fungal community diversity in the leaves, fruits and pneumatophores is comparable, while sediment samples are approximately twice as diverse as all other samples. This is consistent with previous work showing that soils are highly diverse reservoirs of fungal biodiversity, containing hundreds of thousands of fungal species (Bridge and Spooner 2001; Lee et al. 2019a). Likewise we find the highest number of fungal ASVs in the sediment samples. On the one hand, sediments and soils are dynamic environments and the fungi in these environments have diverse roles (Li et al. 2016). On the other hand, the habitats associated with plant organs are expected to be less diverse and more stable, being controlled ultimately by the specific requirements of the host plant. Consequently, fewer fungi can be supported and correspondingly we see a less diverse fungal community associated with non-sediment samples.

As with previous mangrove-associated fungal work, all samples are dominated by phyla Ascomycota and Basidiomycota, and class Dothideomycetes. Class Agaricomycetes is found throughout, but are more prevalent in pneumatophores and sediment, or parts that are inundated at high tide. The Agaricomycetes are frequently found in marine environments and have been reported as one of the dominant fungal classes in tropical mangrove sediments (Arfi et al. 2012; Rédou et al. 2015). The increased prevalence of Agaricomycetes in communities from pneumatophores and sediment that have the potential to be submerged in comparison to those within leaves and fruits likely reflects the adaptations this group has for life in environments where they will be at least partly submerged over a complete tidal cycle (Prasannarai and Sridhar 2001).

Wind and flood events have been proposed as mechanisms that transport terrestrial fungi to mangrove environments (Bonugli-Santos et al. 2015), and air mass source has been shown to be an important determinant of microbial diversity in marine systems (Archer et al. 2019). Results here appear to support these ideas as we have been able to assign taxonomy to the majority of sequences recovered from fruits and leaves, likely reflecting their terrestrial origins and the abundance of mycological work performed in these habitats, for which taxonomic assignments of terrestrial fungi in sequence databases are well curated. Conversely, the highest number of unassigned fungal sequences are found in the pneumatophores and sediment, which are periodically submerged and likely contain a higher proportion of marine fungi (Kohlmeyer 1969). Assigning identities to microbes from marine or understudied environments is an acknowledged challenge as databases curated with marine representatives are lacking (Rédou et al. 2015; Ettinger and Eisen 2019; Archer et al. 2019).

Southeast Asia contains are the most biodiverse, extensive and oldest mangrove forests on the planet (Ellison et al. 1999; Giri et al. 2011; Gandhi and Jones 2019). However, their continued existence faces an uncertain future, with considerable challenges presented by deforestation, aquaculture and a multitude of other anthropogenic stressors (Farnsworth and Ellison 1997; Richards and Friess 2016; Romañach et al. 2018). Restoration and rehabilitation are important mangrove conservation strategies (Renzi et al. 2019; Lee et al. 2019b). Terrestrial restoration schemes frequently incorporate information about fungal communities in their approach (Moora et al. 2004; Quoreshi 2008; Chaudhary et al. 2019), and increasingly, marine conservation initiatives are considering the beneficial properties of microorganisms (Peixoto et al. 2017, 2019; Rosado et al. 2019). Our results show that fungal communities can be differentiated by location, suggesting that these communities have evolved to the plants, specific requirements in each environment. If this is indeed the case, it may be necessary to consider the fungal communities in restoration schemes, especially since host-pathogen resistance can be increased by matching host fungal communities as closely as possible to areas where the host is known to be healthy (Zahn and Amend 2017). These considerations are likely even more important and necessary to avoid maladaptation when mangrove propagules are grown in large ex situ nurseries and outplanted. We recommend that, where feasible, ex situ nurseries should be located as close as possible to the restoration site; findings here suggest that doing so will increase the similarities in fungal community composition between nursery and restoration sites.

## CONCLUSION

There is growing appreciation for the role microorganisms play in all aspects of ecosystem functioning, and the success of mangrove restoration projects is expected to benefit from further detailed characterisation of the mangrove microbiome. This study provides foundational data on the fungal communities associated with various compartments of the mangrove *Sonneratia alba* and more broadly contributes to better understanding of the coastal microbiome. However, more concerted and coordinated cross-disciplinary efforts are required from marine, terrestrial and atmospheric microbiologists to fully address the acknowledged gaps in research of this nature.

## Supplementary information

**Additional file 1: SI Figure 1.** Rarefaction curves showing asymptote was achieved for all samples. **SI Figure 2.** NMDS plot coloured by sampling site location, symbols represent plant part DNA was extracted from. **SI Figure 3.** Shannon diversity for each structure, all locations combined. **SI Figure 4.** Barplot showing the various phyla at each location, all structures combined. **SI Figure 5.** Shannon diversity for each location, all structures combined. **SI Figure 6.** Barplot of fungal class by location, all samples combined by loaction. **SI Figure 7.** Heatmap of class-level taxa distributed through each structure. Deeper red indicates higher abundance. **SI Table 1.** Details of sample size from each location. **SI Table 2.** PERMANOVA showing that location and sampled structure (leaf, fruit, pneumatophore or sediment) significantly influence fungal communities.

## Data Availability

The datasets generated during and/or analysed during the current study are available in the National Center for Biotechnology Information under the BioProject ID PRJNA592423.
